# Large-scale directional relationship extraction and resolution

**DOI:** 10.1186/1471-2105-9-S9-S11

**Published:** 2008-08-12

**Authors:** Cory B Giles, Jonathan D Wren

**Affiliations:** 1 Arthritis and Immunology Research Program, Oklahoma Medical Research Foundation, 825 N.E. 13th Street, Oklahoma City, Oklahoma 73104-5005, USA

## Abstract

**Background:**

Relationships between entities such as genes, chemicals, metabolites, phenotypes and diseases in MEDLINE are often directional. That is, one may affect the other in a positive or negative manner. Detection of causality and direction is key in piecing pathways together and in examining possible implications of experimental results. Because of the size and growth of biomedical literature, it is increasingly important to be able to automate this process as much as possible.

**Results:**

Here we present a method of relation extraction using dependency graph parsing with SVM classification. We tested the SVM classifier first on gold standard corpora from GENIA and find it achieved 82% precision and 94.8% recall (F-measure of 87.9) on these standardized test sets. We then applied the entire system to all available MEDLINE abstracts for two target interactions with known effects. We find that while some directional relations are extracted with low ambiguity, others are apparently contradictory, at least when considered in an isolated context. When examined, it is apparent some are dependent upon the surrounding context (e.g. whether the relationship referred to short-term or long-term effects, or whether the focus was extracellular versus intracellular).

**Conclusion:**

Thesaurus-based directional relation extraction can be done with reasonable accuracy, but is prone to false-positives on larger corpora due to noun modifiers. Furthermore, methods of resolving or disambiguating relationship context and contingencies are important for large-scale corpora.

## Introduction

Large-scale or systems-wide analysis of relationship networks (e.g. protein-protein) is built from individual links. Since labor is expensive, the domain of knowledge vast, and time short, automated methods of constructing these networks is of paramount importance in bioinformatics research [1]. The simplest method of constructing such a network is by co-occurrence of terms, either in sentences or abstracts [2-4]. Previous research found that entities co-occurring within a sentence have approximately an 80% chance of being related in a non-trivial manner, while entities co-occurring within an abstract have approximately a 50% chance [5,6] (exact numbers vary). However, these co-occurrence based approaches, despite their computational efficiency, necessarily remain agnostic about the nature of the relationship between entities. And if we are to have any hope at understanding how control and cause/effect are propagated in these networks, we must establish directionality.

### NLP systems and biological networks

Establishing relationship directionality usually requires some means of natural language processing (NLP) to extract relationships from biomedical text, although it is not necessarily the only way [7,8]. Most systems that attempt to characterize the nature of the relationship between entities (e.g., directionality, stimulation, inhibition) use grammatical information from sentences, which is provided by NLP software. These may use either shallow parsing [9], which divides the sentence into chunks, or deep parsing [10,11], which provides a complete representation of a sentence's constituent grammatical relations. For example, Chilibot [12] and MedScan [13-15] attempt to parse sentences to detect the nature of relationships and develop association networks. Chilibot has only three relationship types – positive, negative, and neutral – while MedScan has many more. Both Chilibot and MedScan attempt to determine which terms are subject and object. Systems can also be distinguished by whether they use grammatical information in a rule-based manner, like Chilibot, or whether they use machine learning techniques, such as SVMs [16].

NLP-based approaches have seen heavy use in identifying protein-protein interactions within text [17-23], gene-disease relationships [24], and have been successful in bolstering and partially reconstructing regulatory networks using text [25]. The goals for these programs range from a simplified two-class method of labeling extracted relationships (e.g., 'stimulates' (positive) or 'inhibits' (negative)), to detection of several dozen semantic types of relationship, such as 'binds', 'cleaves', 'phosphorylates', etc. Our interests in this line of research are to detect paths of directional inference (e.g. A increases B and B decreases C, therefore A decreases C) and their reliability. To accomplish this we must first develop a method that is scalable. Accuracy is desirable, of course, but if an algorithm has a modest accuracy yet many chances to detect directional relations, then it is possible the large sample size may offset a less-than-optimal accuracy. To do this, however, we need to better understand how these methods perform on large unstandardized corpora and the amount of apparently contradictory relationships that are detected as the amount of text is increased and false-positives accrue. Most approaches to date have unfortunately focused on relatively small corpora and it is not clear how they would perform on large datasets (e.g. millions of records).

We anticipate some contradiction will be factual in nature (e.g. one author reporting A increases B and another reporting A decreases B), but most likely the primary form of contradiction will be context-dependent. That is, the context of the relationship may enable both scenarios to be true and therefore the different facts extracted from text only appear to be contradictory when examined in isolation. We also hypothesize that some objects will better serve as subjects of study, which will bias observations towards one type of directional relationship when, in theory, both might be considered equally logical. For example, the relationship between insulin and glucose will tend more towards reports on the extracellular effects of insulin on glucose (insulin decreases glucose) rather than the intracellular effects (insulin increases glucose) because the extracellular effects are much easier to assay for (e.g. by drawing blood). This is going to introduce an artifact into any NLP analysis that will eventually need to be resolved in the thrust towards a more "systems-level" view of biology. For now, however, we hope that such contextual biases will be relatively uniform (e.g. if insulin increases or decreases other entities, the same perspective biases will apply for them) so that making inferences might be possible.

Here we present a directional relation extraction (DRE) system that uses a support vector machine (SVM) to classify dependency paths, as SVMs have been shown recently to be suited to this type of task [26]. We term it the directional relation extraction and determination with SVM (DREAD SVM). It has the following notable features: First, it has a robust, extensible dependency path convolution kernel capable of extracting and determining directionality for relations when trained on the GENIA event corpus. Second, it has a thesaurus-based named entity recognition algorithm that is not limited to a particular type of biomedical entity (such as gene or protein). We then present the results of cross-validation on the GENIA event corpus and explore the results of large-scale extraction from MEDLINE abstracts. We observe that relationships are often extracted with a degree of ambiguity in their direction and nature – a phenomenon that is often viewed as inconvenient noise that is to be screened out during construction of biological networks. However, it is these instances we are most interested in for this report. The construction of biological networks often is centered upon the arrow and node (or vertices and edges) concept. Yet, how many of these relationships are of such a simple nature that they can be represented in such a manner and how many might be essentially irresolvable without a 3^rd ^variable, such as perspective or time? We do not believe we can answer this conclusively here, but through the analysis of several terms expected to have relatively straightforward relationships, we hope to gain a better understanding of how often these simplified relationship constructs are valid.

## Materials and methods

**Figure 1:**
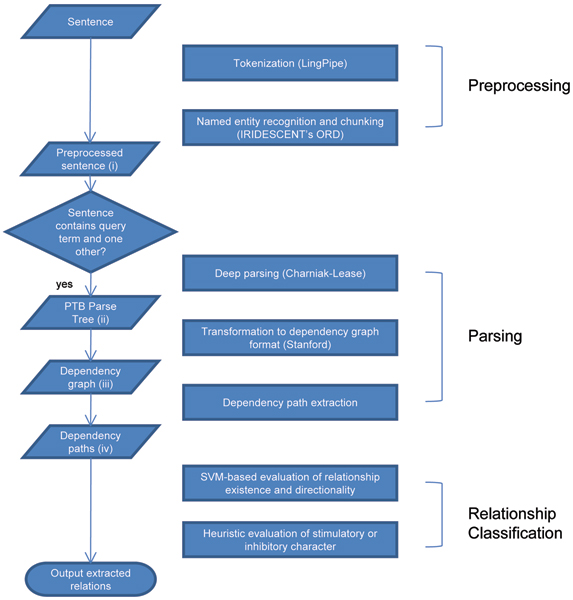
Flowchart of the relation extraction process.

The DREAD algorithm begins with the input of a sentence containing a term of interest, which must appear in a thesaurus of biomedical objects, including genes, chemicals, and clinical phenotypes (described below). The final output of the algorithm is a set of directed edges between the query term and all other terms that co-occurred with the query term within a sentence. These directed edges are also characterized for the presence of stimulatory or inhibitory interactions. In order to achieve this output, the DREAD algorithm goes through several stages of activity: namely Preprocessing, Parsing, and Relationship Resolution (Figure 1). Each stage has a corresponding broad function:

• The *Preprocessing *phase formats the sentence for parsing by tokenizing and Named Entity Recognition (NER).

• The *Parsing *phase uses Natural Language Processing tools to construct a grammatical representation of the sentence in Stanford Dependency (SD) format. The SD format casts words (tokens) as a network of nodes connected by grammatical *dependencies *(edges).

• The *Relationship Classification *phase uses a combination of Support Vector Machines and heuristics to deduce the directionality and possible stimulatory or inhibitory character of a relationship based on the grammatical information provided by the parsing phase.

### Preprocessing

The LingPipe software first breaks each sentence into a sequence of tokens . Next, NER identifies biologically relevant terms within the sentence using a synonym dictionary of genes, chemicals, and clinical phenotypes developed as part of the IRIDESCENT system [6,27,28]. Multiple-token terms are condensed to a single token in order to simplify the subsequent parsing steps. At this point, if the sentence does not contain at least the query term and one other named entity within the synonym dictionary, it is thrown out.

### Parsing

#### Deep parsing

**Figure 2:**
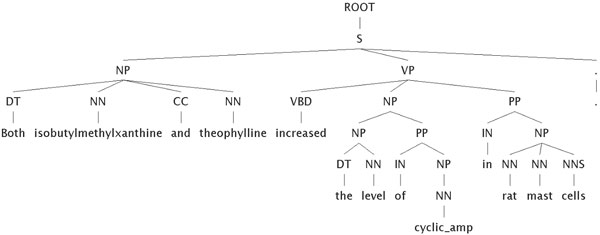
An example of sentence structure for the sentence "*Both isobutylmethylxanthine and theophylline increased the level of cyclic AMP in rat mast cells*.". Shown are the grammatical relationships diagrammed in the Penn Treebank format.

The preprocessed sentences containing entities of interest are sent to the Charniak-Lease parser [29], which performs both part-of-speech tagging and deep parsing, returning a representation of the sentence in Penn Treebank format. The Charniak-Lease parser was previously found to be computationally efficient and accurate on biomedical text compared with other deep parsers [30]. The Penn Treebank (PTB) representation of each sentence parsed is then stored in the local relational database awaiting evaluation by the next steps in the system (Figure 2).

#### Conversion to Stanford Dependency format

**Figure 3:**
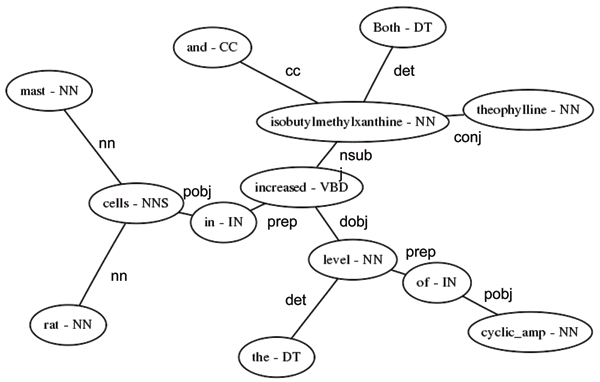
Grammatical sentence structure in dependency graph format.

In order to distill the amount of grammatical information used as feature space for machine-learning-based relation extraction to a small, rich subset, we convert the Penn Treebank-format (PTB) output from the Charniak-Lease parser into the Stanford Dependency (SD) format. This SD format represents sentences as a network of nodes (words) and edges (grammatical relationships between words), as described in de Marneffe *et al *[31] (See Figure 3). In the DREAD system, the PTB->SD conversion is accomplished using a suite of tools available from Stanford; however, recent work has shown that conversion to typed dependencies from other formats, such as the HPSG output of the GENIA team's Enju parser, is also possible [32].

#### Extraction of dependency path

**Figure 4:**
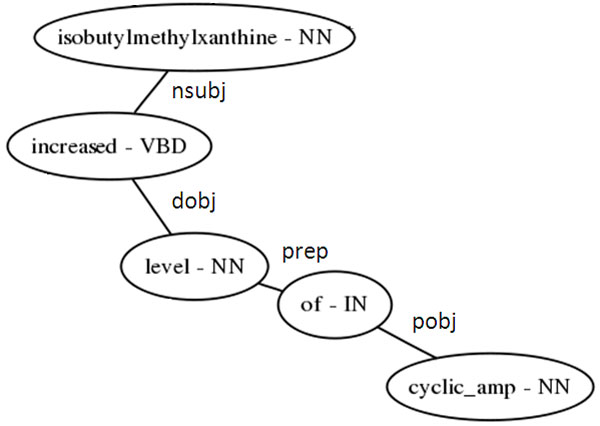
Path of dependency between database terms.

After converting the sentence to SD format, we further reduce the amount of grammatical information used in relation extraction by using only the "dependency path" – that is, the shortest sequence of nodes and edges between two entities in the grammatical network of typed dependencies (Figure 4). The DREAD system extracts a dependency path between the query term and each other term in the sentence that was identified by the dictionary-based NER step. If there are multiple instances of the query term within the sentence, this process is repeated, omitting dependency paths connecting the query term to another instance of itself.

The dependency path has previously been shown to provide a good feature space for machine-learning-based relation extraction [33,34]. Others, however, have used sub-trees of the syntactic parse tree (i.e., Penn Treebank format) or of the typed dependency format as feature space for relation extraction, to good effect [35]. A study of the optimal feature space for relation extraction is provided in Jiang and Zhai [36].

### Relationship classification

**Figure 5:**
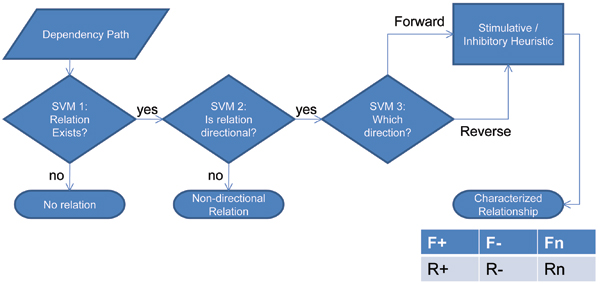
SVM identification of directional relationships.

In order for the DREAD system to draw a conclusion about the nature of a relationship based on the grammatical information provided by a dependency path, we use two sub-systems. The first, a pipeline of Support Vector Machines (SVMs), is used to establish the presence and directionality of a relationship for each dependency path. If the SVM classification procedure determines that the dependency path does indeed represent a directional relationship, the second system, a simple set of heuristic rules, classifies the relationship as "stimulatory" (+), "inhibitory" (-), or "neutral" (n). Together, these two pieces of information (directionality and stimulatory/inhibitory classification) are a summarized form of the interaction between two entities in the sentence (Figure 5).

#### SVM-based directionality determination

The SVM classification pipeline is composed of three one-against-one "convolution dependency path kernel" SVMs (described below). Each SVM has a training set derived by parsing and transforming the GENIA event corpus [37,38] into dependency paths. The three SVMs classify the dependency path in the following ways:

1. Is there a functional relationship between the two entities?

2. If there is a relationship, is that relationship directional?

3. If directional, is the direction forward or reverse?

If SVM #1 or #2 returns a negative answer (i.e., there is no relationship or the relationship is not directed), then the dependency path is not sent to any further steps.

#### Convolution dependency path kernel

Bunescu described the development of a SVM kernel that computes the similarity of two sequences of features by the number and length of common subsequences between them [33]. In that work, however, Bunescu used tokens, part-of-speech tags, and entity classes in the order that they appear within the sentence. As a concrete example, in comparing the sentences "I went to the store" and "You went to the game", the longest common subsequence of tokens would be "went...the", and other subsequences would be "went...to" and "to...the". In another work, Bunescu *et al *restricted feature classes to those within dependency paths, but simply calculated the number of co-occurring features as the kernel score [39]. This method had the limitation that paths with different lengths were computed as having zero similarity, which presumably lowered recall.

We reasoned that the accuracy of the overall SVM procedure could be improved by combining the two methods; in other words, by restricting the set of features to those within the dependency path but by using Bunescu's subsequence kernel to compare two potential relationships with greater flexibility. Indeed, a recent study by Wang, who coined the name "convolution dependency path kernel", showed that combining the two methods leads to greater precision and recall [40].

Formally, the generalized subsequence kernel is defined [39]:

$$K_{n}(s,t,\lambda )=\sum_{u\in \Sigma_{\cup }^{n}}\sum_{i:u≺s[i]}\sum_{j:u≺t[j]}\lambda^{l(i)+l(j)}$$

Where *u *is a subsequence from the set of all possible sequences $\left(u\in \Sigma_{\cup }^{n}\right)$ subject to the constraint that *u *must appear as a subsequence in both of the feature sequences *s *and *t *(that is, *u *≺ s[i], u ≺ t[j]); **i **and **j **are the sequences of indices corresponding to *s *and *t*. Each subsequence *u *is penalized according to the decaying factor, λ, with the result that when the feature sequences *s *and *t *are longer, each subsequence is given less weight. This serves as a way of normalizing the kernel scores for feature sequences of different lengths.

#### Feature selection

There are three primary types of feature available in each dependency path for use by machine-learning classification: the token (word), the part-of-speech tag for each token, and the grammatical dependency (SD edge) connecting two tokens. We elect not to use the token as a feature; because MEDLINE abstracts consist of such a large vocabulary, choosing the token would likely lead to overfitting of the training data. On the other hand, both POS tags and grammatical dependencies provide a relatively sparse set of feature classes, and are more relevant to the grammatical structure of the sentence than the token itself.

### GENIA training corpus

The GENIA event corpus consists of 1,000 abstracts from MEDLINE that have been manually annotated for various biomedical "events", largely molecular interactions. Of the events in this corpus involving two entities, some are directed, while others are not. These events have various annotations; we considered all relations of the *Positive_regulation *type as stimulatory, all relations of the *Negative_regulation *type as inhibitory, and all others as neutral. To derive gold standard relationships from the corpus, we first assembled all possible pair-wise combinations of entities within the sentence (because the GENIA event corpus is in a quasi-XML format, there are sometimes entities within entities; the broadest possible entity names were used). These pairs were then classified as follows:

1. Exists vs. nonexistent – If an event was annotated between a pair of entities, a functional relationship between the two entities was said to exist; otherwise, the pair was classified as non-interacting.

2. Directional vs. non-directional – Of the pairs of entities that were annotated as interacting, some relations showed one entity acting on the other (in GENIA parlance, this relationship had one "THEME" and one "CAUSE"), while other relationships did not show directionality (two "THEME" annotations). We divided interactions on this basis for the second training set.

3. Forward vs. reverse – For directional relations, the "CAUSE" was considered to be the agent of the interaction, while the "THEME" was the target.

To complete construction of the training corpus, we parsed all sentences and extracted dependency paths between each pair of entities as previously described. Dependency paths from MEDLINE at large could then be compared by the SVM kernel to dependency paths within the training corpus to classify the existence and directionality of relationships through a series of one-against-one classifiers.

#### Heuristic determination of stimulatory or inhibitory relationships

After using the pipeline of SVMs to establish the presence or absence of a relationship and, if applicable, its directionality, we apply a simple set of heuristic rules to determine whether the nature of the relationship described by the dependency path is "positive" (stimulation, induction, activation), "negative" (repression, down-regulation, deactivation), or "neutral" (none-of-the-above). This is done by matching stemmed tokens in the dependency path to a list of words in each category. We hope to soon characterize the stimulatory or inhibitory nature of relationships using a SVM [16]; however, initial efforts using tokens as features have resulted in good cross-validation accuracy albeit poor recall in large-scale MEDLINE extraction.

### Relationship resolution on large-scale datasets

In order to test the performance of the DREAD algorithm on a large scale, and to test the hypothesis that aggregating relationship data from a large corpus will overcome deficiencies in precision on individual sentences, we sought out summary information for different objects in the database. We chose two common terms in the biomedical literature to focus on for analysis of unstructured text: A chemical, caffeine (17,145 papers with this term in the title and/or abstract) and gene, *c-myc *(11,971 papers). Summary information regarding the direction and nature of relationships between caffeine and other objects in the database was compiled from its Wikipedia entry . For c-myc, we used NCBI's Gene Reference into Function (GeneRIF)[41] instead because there was more information. Although Wikipedia may not always be appropriate as a source of summary information, we did feel that analysis of system performance should optimally be constrained to one or two standard sources of summary information so as not to bias the analysis (e.g., noticing that the system worked really well for a certain term and then hunting down supporting information related to that term while ignoring other terms that the system performed poorly on).

After thus compiling a list of expected relationships between the two terms and other biomedical objects, we pulled all abstracts containing caffeine and *c-myc *from MEDLINE, split each abstract into sentences using the LingPipe utility, and preprocessed each sentence as described above. If the sentence was found to contain the target term, the DREAD system was used to predict the relationships between the target term and each other term in the sentence. If a directional relationship was found, it was categorized as either "forward" (F), indicating that caffeine or *c-myc *affects the other object, or as "reverse" (R), indicating that the other object acts upon caffeine or *c-myc*. "+" is defined as a stimulatory (e.g. increases, raises, upregulates, etc) relationship between two terms and "-" is an inhibitory relationship (e.g. decreases, lowers, inhibits, downregulates, etc.). For example, the relationship "caffeine increases cyclic AMP" would be notated "F+" from the perspective of caffeine.

These relationships were then aggregated and each binary relationship was assigned a strength score based on the amount of contradiction. Extracted relations were considered contradictory when both stimulatory and inhibitory instances were detected for the same directionality. For example, finding A increases B and A decreases B results in a contradiction, but finding A increases B and B decreases A is not necessarily contradictory. Feedback and feedforward loops, for example, can accommodate such trends. Forward scores are calculated as max(F+, F-)/((F+)(F-)) and reverse scores as max(R+, R-)/((R+)(R-)).

## Results

### SVM cross validation on GENIA corpus

**Figure 6:**
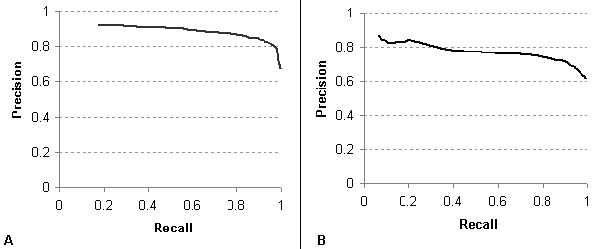
Precision/recall curve for A) Detecting relationships and B) Detecting directional relationships within the GENIA corpus.

**Figure 7:**
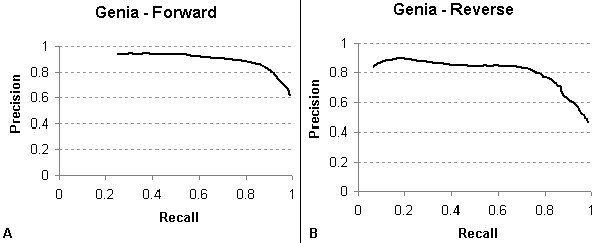
Precision/recall curve for detecting A) Forward relationships and B) Reverse relationships in the GENIA corpus.

To benchmark the performance of the convolution dependency path kernel on standardized biomedical corpora, we subjected the GENIA training data for each of the three SVMs (see *GENIA training corpus *section) to 10-fold cross-validation. For detecting the existence of a relationship and directionality to the relationship, the precision/recall curves are shown in Figure 6. Contrasting with Küffner *et al *[16], the DREAD SVM's ability to detect the existence of a relationship achieved a maximum F-measure of **0.879 **(Küffner 0.849) at a precision level of **0.820 **(Küffner 0.827) and recall of **0.948 **(Küffner 0.872). The ability of our algorithm in directional relationship extraction (DRE) achieved a maximum F-measure of **0.794 **(Küffner 0.749) at a precision level of **0.704 **(Küffner 0.846) and recall of **0.912 **(Küffner 0.672). For directional relationships, the precision/recall curves are shown in Figure 7. F-measure scores were similar between detection of forward (0.861) and reverse (0.791) relationships.

Note that these scores are based upon the evaluation of dependency paths in which the recognition of named entities is part of the corpus; therefore, they do not take into account the considerable loss in recall and slight loss in precision that results from a dictionary-based named entity approach. Also, in the DREAD system, these SVMs are arranged in a pipeline fashion, so that the error rate propagates in a multiplicative manner. Thus, these scores are a meaningful marker only of the performance of the SVM classifiers and not of the DREAD system as a whole.

### Method scalability

For comparable tasks, DREAD SVM performance on the GENIA corpus was comparable to levels of precision and recall reported by Küffner *et al *[16]. This suggested that it was reasonable to proceed forward and examine how well performance would scale on much larger corpora.

#### Caffeine analysis

Using summary information for each of our target terms to know what relationships should be found, and the nature of those relationships, we ran the DREAD SVM on all MEDLINE abstracts with the target term. The same directional relationship had to be observed at least twice to be counted as a directional relationship prediction. Table 1 summarizes the DREAD SVM performance on extracting directional relationships between caffeine and other objects in MEDLINE. Caffeine was chosen for three reasons: First, it was expected to have a predominance of stimulatory relationships. Second, it was expected to affect other objects in the body, but not be affected by many other objects (outside of metabolic pathways). And third, the authors have substantial first-hand experience with the physiological effects of this chemical compound. Table 1 shows that most relationships are indeed forward (253 versus 116 reverse) and stimulatory (118 versus 44 inhibitory), as expected, but clearly not every extracted directional relationship was unambiguous. The system only identified 12/21 (57%) of the expected relationships. It did, however, correctly identify the directionality of 10/12 (83%) of the relationships.

**Table 1:** Identifying directional relationships in MEDLINE for the chemical compound caffeine on the basis of summarized relationships after analysis of 17,145 abstracts.

**ObjectName**	**No rel.**	**ND Rel.**	**F+**	**F-**	**Fn**	**R+**	**R-**	**Rn**	**Expected**	**Extracted**
(-)-Adrenaline	58	22	14	4	17	0	3	17	F+	F+
(-)-Dopa	5	2	1	0	1	1	0	2	F+	None
Cyclic AMP	30	9	21	11	12	4	3	13	F+	F+
diuresis	0	0	2	0	0	0	0	0	F+	F+
***Drowsiness***	***2***	***0***	***3***	***1***	***1***	***1***	***0***	***0***	***F*-**	***F+***
Fatigue	23	8	4	6	15	1	0	11	F-	F-
Fatty acid	10	0	1	0	6	0	0	2	F+	None
gastric acid secretion	3	3	1	0	2	0	0	1	F+	None
***Glycerol***	***15***	***6***	***2***	***3***	***8***	***0***	***0***	***3***	***F+***	***F*-**
insomnia	16	3	3	0	5	0	0	2	F+	F+
Irritability	2	2	2	1	1	0	0	1	F+	F+
Lethargy	2	0	0	1	0	0	0	0	F-	None
lipid metabolism	0	0	0	0	1	0	0	2	F+	None
Nervousness	0	1	0	1	5	0	0	1	F+	None
Norepinephrine	61	19	10	2	18	4	0	16	F+	F+
Palpitation	5	1	0	0	1	0	0	2	F+	None
PKA	3	1	1	0	3	0	0	2	F+	None
Respiratory alkalosis	0	0	0	0	1	0	0	0	F+	None
ryanodine-sensitive calcium-release channel activity	48	18	33	2	17	5	4	15	F+	F+
vasodilation	2	0	1	2	1	0	0	0	F-	F-
vasoconstriction	5	0	3	0	2	0	0	0	F+	F+

Possible directional relationships are classified as **F**orward (caffeine affects the object) or **R**everse (the object affects caffeine) and as stimulatory (+), inhibitory (-) or neutral (n). No rel = no relationship found, N.D. rel = no directional relationship found, Expected = the directional relation suggested by the summary information, Extracted = the directional relation with the most support, based on the sentences processed. ***Bold italic ***font indicates errors.

Drowsiness would naturally seem like a phenotype that should be lowered upon caffeine intake. However, caffeine withdrawal can cause drowsiness [42] and since "caffeine withdrawal" was not present in the database, the shorter term was recognized as the subject instead.

#### C-myc analysis

The oncogene, c-myc is well studied and expected to both affect other objects and in turn be affected, as part of a genetic network. The mechanisms which sometimes govern genetic control, especially over a gene that is estimated to possibly regulate up to 15% of all genes[43] are expected to complicate DRE. Table 2 summarizes the results. The system identified 21/39 (53%) of the expected relationships, and of those, it correctly identified the directionality for 13/21 (62%). Examining some of the individual sentences to better understand the reasons for failure, it became evident that the natures of relationships for c-myc were less straightforward than for caffeine. For example, the role of c-myc as an oncogene is well known and it is upregulated in breast cancer, yet the system identified many more examples of breast cancer "stimulating" c-myc. This is because these examples are reported as correlative relationships (e.g., "in the breast cancer biopsy, c-myc was upregulated").

**Table 2:** Identifying directional relationships in MEDLINE for the gene c-myc.

**ObjectName**	**No rel.**	**ND Rel.**	**F+**	**F-**	**Fn**	**R+**	**R-**	**Rn**	**Expected**	**Extracted**
*Process*										
Anaplasia	6	1	0	0	1	1	0	4	F+/R+	None
***Angiogenesis***	***11***	***10***	***1***	***2***	***7***	***1***	***1***	***3***	***F+***	***F*-**
***Breast cancer***	***92***	***12***	***2***	***0***	***44***	***9***	***2***	***40***	***F+***	***R+***
Cell growth	131	34	19	19	76	6	8	34	F+	Inc.
Cervical carcinoma	22	4	0	0	3	0	0	8	F+	None
***DNA Damage***	***33***	***7***	***1***	***0***	***14***	***3***	***2***	***11***	***F+***	R+
DNA repair	13	11	0	0	1	1	0	4	F-	None
Medulloblastoma	21	6	4	0	13	1	0	20	F+	F+
Tumorigenesis	80	40	20	6	48	6	4	32	F+	F+
										
*Chemical*										
Rapamycin	4	0	1	0	2	0	1	0	R+	None
Reactive oxygen species	9	5	2	0	4	1	1	10	F+	F+
Valproic acid	1	0	0	0	0	0	2	0	R-	R-
										
*Gene*										
AICDA	4	0	0	0	0	0	0	1	R+	None
AURKA	5	1	0	0	2	0	0	1	R+	None
Calcineurin	0	0	0	1	0	0	0	0	R+	None
***CDKN2A***	***95***	***37***	***5***	***2***	***26***	***1***	***7***	***42***	***F*-**	***R*-**
CREBBP	7	3	0	0	5	1	0	6	R-	None
EPHA2	8	0	1	0	1	0	0	2	F-	None
***FBXW7***	***18***	***5***	***0***	***0***	***4***	***4***	***1***	***17***	***R*-**	R+
HDAC1	1	5	0	0	0	0	0	4	F+	None
HMGCS2	1	0	0	1	0	0	0	1	F-	None
IFN-gamma	2	1	1	0	0	0	1	1	R-	None
***JUN***	***558***	***28***	***12***	***0***	***71***	***12***	***6***	***75***	***F*-**	F+/R+
NDRG2	2	1	0	0	5	0	0	2	F-	None
NFATC1	1	0	0	0	1	2	0	1	R+	R+
PCGF2	4	4	0	0	0	0	0	2	R-	None
PPARG	1	1	0	0	1	0	0	2	F+	None
PRL	15	0	0	0	5	6	1	5	R+	R+
***RAC1***	***5***	***3***	***2***	***1***	***1***	***0***	***0***	***2***	***R+***	F+
SP1	29	5	0	3	9	4	0	9	R+	R+
***STAT5A***	***6***	***2***	***1***	***0***	***0***	***2***	***0***	***4***	***R*-**	R+
Telomerase	27	5	13	0	9	5	1	8	F+	F+
VEGFA	49	2	0	1	18	0	0	17	F+	None
WRN	1	1	2	0	3	0	0	2	F+	F+
ZBTB16	3	2	0	0	0	0	0	1	R-	None
ZBTB17	24	17	1	8	11	1	3	9	F-	F-
Zfp472	1	2	0	0	0	0	0	1	R-	None

See Table 1 for header explanations. ("Inc." = inconclusive - tied for the highest relation score).

Another example is JUN (aka c-jun). The actual functional relationship between c-myc and JUN is forward, negative: c-myc inhibits c-jun [44]. However, because both c-myc and c-jun are oncogenes, they are usually mentioned in the context of being upregulated together. Yet another difficulty in the analysis of c-myc is that sentences often refer to an increase or decrease in the effects or properties (e.g. "oncogenic properties") of c-myc, rather than c-myc itself. Table 3 shows examples of sentences whose DRE was incorrectly classified. There is an average of 0.11 stimulatory terms per c-myc:c-jun path, but only 0.03 inhibitory terms. So, in this case, at least, there seems to be a literature bias towards describing relationships as stimulatory rather than inhibitory. We examined the different interaction types to see if any were longer on average or contained more stimulatory or inhibitory keywords, finding that gene-gene candidate interaction paths were considerably shorter than gene-process paths, and contained fewer interaction keywords (see Table 4).

**Table 3:** Example sentences from the failed DRE between c-myc and the entities "Breast Cancer" and "c-jun". The expected relationships for breast cancer was F+ and for c-jun, F-.

Object	Extracted Rel.	PubMed ID	Sentence
Breast Cancer	R+	12150449	The c-myc oncogene is frequently activated in invasive breast cancer and has been associated with high nuclear grade, lymph node metastasis and poorer disease outcome
	R+	7734397	The proto-oncogene c-myc is involved in the stimulation of cell proliferation, and its expression is known to be stimulated by estradiol (E2) in human breast cancer cell lines and various non – cancerous E2 – dependent tissues
	F+	1855215	In search of critical genes in the mechanism of estrogen action in human breast cancer, we previously showed that estrogen stimulates transcription of the c – myc gene in estrogen-dependent (MCF-7) cells
			
c-Jun	R+	8417822	17 beta – Estradiol had little effect on expression of c-jun, jun B, jun D, or c-fos mRNA by MCF-7 cells over 12 h, although it stimulated c-myc expression 4-fold within 30 min
	F+	14523011	Furthermore, we identify a phylogenetically conserved AP-1-responsive element in the promoter of the c-myc proto-oncogene that recruits in vivo the c-Jun and JunD AP-1 family members and controls the PDGF-dependent transactivation of the c-myc promoter
	R-	8219202	In addition, intracellularly, mitoxantrone-induced PCD was associated with a marked induction of c-jun and significant repression of c-myc and BCL-2 oncogenes

**Table 4:** Path lengths and quantity of stimulatory and inhibitory tokens seem to vary with the type of object.

	**Number of Paths**	**Average Path Length (tokens)**	**Average stimulatory tokens per path**	**Average inhibitory tokens per path**
**Myc:chemical**	42	7.64	0.36	0.21
**Myc:process**	850	7.23	0.16	0.11
**Myc:gene**	1378	4.93	0.15	0.06

See Table 2 for the particular objects tested.

## Discussion and conclusion

Analysis of unstandardized text comes with many challenges for term recognition, synonym mapping, homonym resolution, etc. And directional relation detection is yet another challenge, not just in a technical sense of algorithmically detecting relations, but also in the sense of resolving apparently contradictory data that may arise. NLP approaches so far isolate facts from sentences and resolve relationships between two entities, which may not be sufficient to accurately reconstruct or model relationship networks. As we report here, extracted relationships range from those with strong support for one type and direction of relationship over another to apparently contradictory relationships, some of which are quickly resolved by a better understanding of context.

We find that for these tasks involving directional relation extraction, it is important to have terms be as unambiguous as possible. Stress and metabolism, for example, have common meanings (i.e., psychological stress and the rate of energy consumption by an organism, respectively) and more specific physiological meanings (i.e., a state of increased responsiveness to stimulus and the process by which chemicals change form, respectively). One approach to increase DRE accuracy would entail employing methods to break down broad categories into more specific subcategories (e.g. oxidative stress, physiological stress, stress-related illness, etc.), as the recognition of term relationships was not the limiting factor in this analysis but rather the accurate recognition of full terms.

We also find that noun modifiers frequently complicate thesaurus-based analysis of terms, as in the case of c-myc. To increase or decrease a gene in a biological sense would likely refer to its mRNA levels, protein expression levels or molecular activity (e.g. catalytic) levels. However, study of genetic effects often proceeds via an assessment of how known function is affected by manipulating the system and thus increasing and decreasing statements refer to the modifier rather than the gene (e.g. "A decreased gene-B-related apoptosis", "A increased geneB phosphorylation", etc). While NLP systems are capable, in theory, of resolving such sentences, this style of study and writing causes more false-positives as corpus size increases.

Ultimately, the scientific community is going to want to move towards testing of extracted networks for concordance with observed behavior. But to do this, a greater incorporation of contextual or conditional information will become necessary, which may not be possible to represent in a single graph. Temporal effects, for example, would seem to require a graph for short-term and long-term effects. Additionally, some model organisms are manipulated to alter genetic behavior, and relationships that are true within a mutant strain may not be true outside it. Similarly, some interactions are conditional. Worse, some of these conditional interactions may show up as contradictory relationships merely because not enough is known to understand that "A increases B" and "A decreases B" are both true depending upon the context. All this unfortunately complicates analysis, but at the same time seems unavoidable. Networks may well need several additional parameters per connection to accommodate contextual information. In part, this type of NLP work may guide some of this along – when apparently contradictory information is found, it is possible to initiate a secondary search for contextual clues that could accurately predict when one relationship type would be true over another. In such cases, a computer need not necessarily "understand" the context, but simply be able to identify it algorithmically. In the future, we hope to develop more accurate metrics to identify when directional relationships require context resolution prior to their inclusion in a biological network.

## Competing interests

The authors declare that they have no competing interests.

## Authors' contributions

JDW conceived of and supervised the project, CBG wrote the programs and implemented them. Both authors contributed to writing the manuscript.
